# *In Vivo* Regulation of Erythropoiesis by Chemically Inducible Dimerization of the Erythropoietin Receptor Intracellular Domain

**DOI:** 10.1371/journal.pone.0119442

**Published:** 2015-03-19

**Authors:** Norio Suzuki, Harumi Y. Mukai, Masayuki Yamamoto

**Affiliations:** 1 Division of Interdisciplinary Medical Science, Center for Oxygen Medicine, United Centers for Advanced Research and Translational Medicine, Tohoku University Graduate School of Medicine, Sendai, Japan; 2 Department of Medical Biochemistry, Tohoku University Graduate School of Medicine, Sendai, Japan; Institut national de la santé et de la recherche médicale (INSERM), FRANCE

## Abstract

Erythropoietin (Epo) and its receptor (EpoR) are required for the regulation of erythropoiesis. Epo binds to the EpoR homodimer on the surface of erythroid progenitors and erythroblasts, and positions the intracellular domains of the homodimer to be in close proximity with each other. This conformational change is sufficient for the initiation of Epo-EpoR signal transduction. Here, we established a system of chemically regulated erythropoiesis in transgenic mice expressing a modified EpoR intracellular domain (amino acids 247–406) in which dimerization is induced using a specific compound (chemical inducer of dimerization, CID). Erythropoiesis is reversibly induced by oral administration of the CID to the transgenic mice. Because transgene expression is limited to hematopoietic cells by the *Gata1* gene regulatory region, the effect of the CID is limited to erythropoiesis without adverse effects. Additionally, we show that the 160 amino acid sequence is the minimal essential domain of EpoR for intracellular signaling of chemically inducible erythropoiesis *in vivo*. We propose that the CID-dependent dimerization system combined with the EpoR intracellular domain and the *Gata1* gene regulatory region generates a novel peroral strategy for the treatment of anemia.

## Introduction

Erythropoietin (Epo) is a 34-kDa glycoprotein that interacts with its specific receptor (EpoR) to regulate growth, differentiation, and survival of erythroid progenitors in response to reduced oxygen delivery [1, 2]. Epo is produced mainly by renal Epo-producing cells (REP cells) in the kidney in a hypoxia-inducible manner, and its production is reduced in patients with chronic kidney disease [3–6]. Recombinant human erythropoietin (rHuEPO) is widely used to correct anemia associated with Epo deficiency. rHuEPO administration alleviates the necessity for blood transfusions and greatly improves patient quality of life [1]. Although high doses of rHuEPO may be beneficial for patients with chronic anemia, the cost and need for frequent subcutaneous administrations are limiting factors for long-term therapies [7]. Additionally, it has been noted that antibodies against Epo develop following rHuEPO treatment in some cases [8]. Consequently, various strategies and Epo derivatives have been investigated to improve Epo therapy [7]. For example, modification of rHuEPO by additional sugar chains or polyethylene glycol prolongs its half-life in the plasma. Small peptides mimicking Epo have also been found, and possibilities for oral administration are being evaluated in clinical studies.

Epo exerts its biological activity by binding to EpoR, a 66-kDa transmembrane protein [9]. EpoR-null mutant mice show a phenotype identical to Epo-null mice, which exhibit embryonic lethality at approximately embryonic day 13 (E13) due to severe anemia [2]. EpoR belongs to the class I cytokine receptor superfamily, which is characterized by a single transmembrane domain and no intrinsic kinase activity [9, 10]. Two EpoR molecules associate with each other through their transmembrane domains on the surfaces of erythroid lineage cells, and the intracellular domains move into proximity with each other after the binding of one Epo molecule to the receptor homodimer [10, 11]. This conformational change induces JAK2 activation, followed by receptor tyrosine phosphorylation, and the recruitment of signaling molecules, such as SHP1, SHP2, Grb2, PI3K, and STAT5 [9, 10]. Thus, the close proximity of the intracellular domain of EpoR is a key event in Epo-EpoR signaling. A similar signaling system has been adopted for other cytokine pathways, including Tpo, SCF, G-CSF, and FGF [12, 13].

A system has been developed that allows for the reversible control of the homodimerization of intracellular proteins in response to a lipid-soluble dimeric form of FK506, which binds to two molecules of FK506-binding protein (FKBP) fusion proteins [14]. To avoid effects on the endogenous FKBP system, a mutated form of FKBP (DmrB) and its chemical inducer of dimerization (CID) were established. It has been reported that CID induces the growth of cells expressing DmrB proteins fused with the signaling domains of EpoR, c-Mpl (Tpo receptor), c-Kit (SCF receptor), G-CSF-R, or FGF-R [14–20]. These data demonstrate that the CID system can be used to regulate cytokine signaling *in vivo* and *in vitro*. Additionally, these reports show that ligand-receptor specificity is not important for cytokine signaling pathways and that dimerization of the receptor intracellular domain is critical for the regulation of cell fate through the activation of cytokine signaling pathways. However, these previous studies did not clearly investigate cell lineage-specific regulation because the CID targeted a wide range of cells expressing the DmrB fusion proteins from retroviral vectors.

We previously reported that an 8.0-kb genomic fragment of the *Gata1* gene, *G1HRD* (*Gata1* gene hematopoietic regulatory domain), is sufficient for recapitulating the endogenous GATA1 expression profile in erythroid cells [21, 22]. We further showed that the germline EpoR-deficient mice could be rescued from their embryonical lethal anemia by transgenic expression of EpoR under the control of the *G1HRD* promoter [23]. Therefore, *G1HRD* is considered a suitable gene regulatory region to control the expression of DmrB fusion proteins for the establishment of CID-dependent erythropoiesis in transgenic mice.

Because CID is a 1.4-kDa small molecule [15], it is expected that oral administration of CID is feasible. In this study, we show that CID exclusively stimulates erythroid cells upon peroral administration to transgenic mice expressing the DmrB-EpoR fusion protein under the regulation of *G1HRD*. We also suggest that the 160 amino acid sequence derived from the EpoR intracellular domain, which was used here as the DmrB fusion protein, may represent the minimal essential motif for EpoR signal transduction to regulate erythropoiesis *in vivo* under the chemically inducible dimerization system [24].

## Materials and Methods

### Ethical Statement

All procedures in this study *were approved by the* committee of Use of Laboratory Animals in Tohoku University (Permit Number: 2012ido-143) in accordance with the Guidelines on Access to Genetic Resources for Users, and the Guidelines for Proper Conduct of Animal Experiments in Japan. All mice were sacrificed by decapitation after exposure to Isoflurane until their hearts ceased beating, and all efforts were made to minimize suffering.

### *G1HRD-idEpoRic* mice

The homodimer system (Takara, Japan) containing the chemical inducer of dimerization (CID, also called AP20187 or B/B Homodimerizer) and the pHom-Mem1 plasmid were purchased [18]. The intracellular domain of EpoR cDNA (247–406 amino acids) [23] was fused to GFP cDNA (pEGFP-N1; Takara), and the EpoR-GFP cDNA was inserted into the pHom-Mem1 plasmid containing a myristoylation signal peptide and the cDNA encoding the CID-binding protein (DmrB). The resulting product was ligated to a mouse *Gata1* genomic fragment (*GIHRD*) spanning from 3.9 kb upstream of the first exon (IE exon) to the second exon [21]. The transgenic construct was referred to as *G1HRD-idEpoRic* (inducible dimerization of EpoR intracellular domain) and injected into fertilized eggs from BDF1 parents (CREA Japan). All mice were genotyped by PCR amplification of the *GFP* gene [25]. CID (10 mg/kg) and rHuEPO (300 U/kg; Chugai Pharmaceutical, Japan) were administered intraperitoneally. For oral administration, CID (100 mg/kg) was diluted with 50% PEG400 (Wako, Japan) in saline and immediately administered using a gastric tube. More than 3 mice or embryos in each experimental group were used in every experiment.

### RT-PCR

Semi-quantitative PCR of the transgene was performed with primers corresponding to the erythroid-specific first exon (IE exon) of the mouse *Gata1* gene and DmrB cDNA (5’-TCCTCTGCATCAACAAGCCC and 5’-CTCCTCCCATCCCCTAATGAC, respectively). The expression of the hypoxanthine guanine phosphoribosyl transferase (*Hprt*) gene was used as an internal control [23].

### Colony-formation assay

Mononuclear cells (MNCs) were prepared from bone marrow or spleen tissue using Histopaque (Sigma), and 1.0×10^5^ MNCs were cultured in a 3.5-cm dish containing methylcellulose medium (Stem Cell Technologies) supplemented with CID or rHuEPO. After 3 days of culture, the cells were stained with benzidine (Wako), and positive colonies were counted as colony-forming unit-erythroid (CFU-E). To count erythroid burst-forming unit erythroid (BFU-E)–derived colonies, the cells were cultured with 2.0 U/mL rHuEPO and 100 ng/mL stem cell factor (SCF, R&D Systems) for 7 days [23].

### Flowcytometry

MNCs were stained with allophycocyanin (APC)-conjugated anti-Mac1, phycoerythrin (PE)-conjugated anti-Ter119, anti-CD71, or anti-B220 antibodies. APC-, PE-, and FITC-conjugated rat IgG2b antibodies were used as isotype-matched controls. To detect phosphorylated STAT1 and STAT5, permeabilized cells were stained with PE-conjugated specific antibodies against phosphorylated STAT5(Y694) or STAT1(Y701) according to the instructions of the PhosFlow kit (BD Biosciences). All antibodies were purchased from BD Biosciences. The samples were subjected to analysis with a FACSCalibur flowcytometer (Becton Dickinson).

### Blood parameters

Blood was collected from the retro-orbital plexus in tubes coated with EDTA, and blood indices were measured using an automatic counter (Nihon-Kohden, Japan). Peripheral blood smears were stained with methylene blue (Muto, Japan), and the cells containing more than two blue dots were counted as reticulocytes. For the blood biochemical test, the serum was examined using Dri-Chem (Fujifilm Medical, Japan).

### Histological analyses

Spleens were fixed in 4% paraformaldehyde followed by embedding in OCT compound (Sakura Finetechnical, Japan) and quick freezing. To detect idEpoRic, 50-μm sections were observed by laser scanning confocal microscopy (LSM 510, Carl Zeiss). To detect erythroid cells, 8-μm cryosections were stained with a rabbit anti-ß-globin antibody (1:10,000 dilution; Research Plus). Antibody binding was visualized as brown signals using a horseradish peroxidase (HRP)-conjugated anti-rabbit immunoglobulin antibody (1:3,000 dilution; Biosource).

### Statistical analysis

Data are presented as the mean ± standard deviation. *P* values were calculated based on a two-tailed, unpaired Student’s *t*-test.

## Results

### Establishment of transgenic mice expressing idEpoRic under the control of *G1HRD*

**Fig 1:**
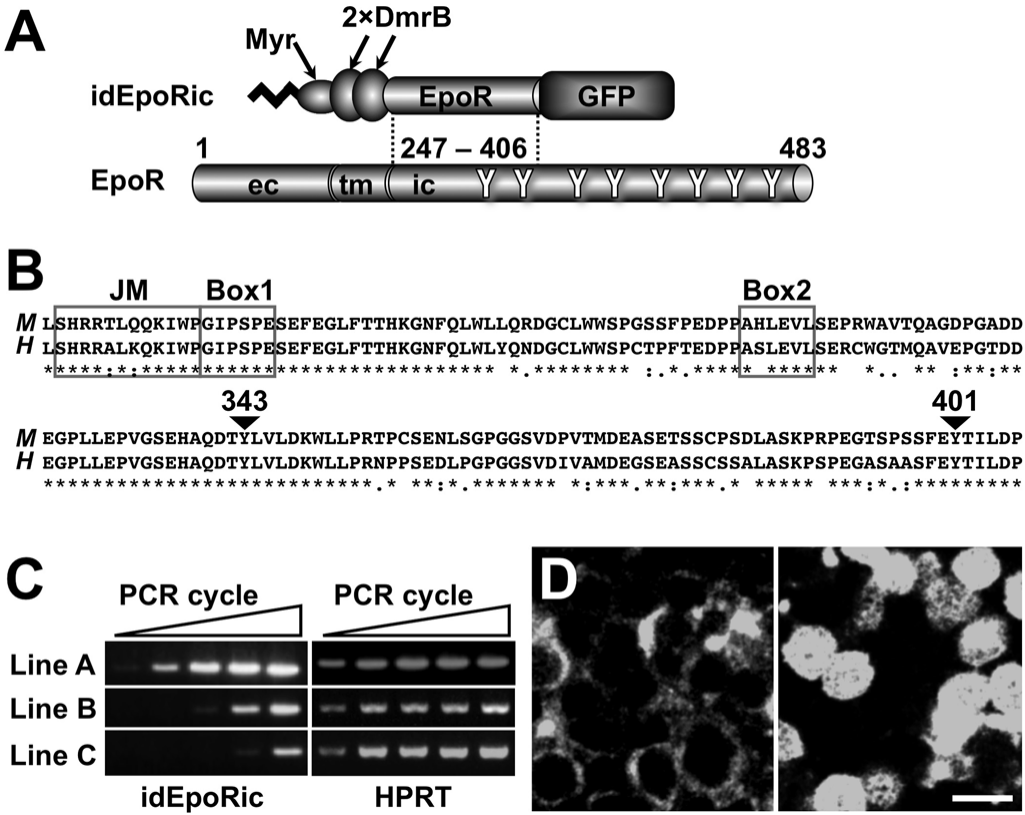
Establishment of *G1HRD-idEpoRic* Transgenic Mouse Lines. (A) Schematic structure of the transgene product, idEpoRic (inducible dimerization of EpoR intracellular domain). The myristoylation signal sequence (Myr) is anchored to the amino terminal of idEpoRic for membrane association with myristate. The EpoR intracellular domain (ic, amino acids 247–406), which contains 2 tyrosine residues (Y), was fused with GFP to monitor the transgene expression. Two copies of the CID binding domain (DmrB) were inserted. The terms ec and tm indicate the extracellular domain and transmembrane domain of EpoR, respectively. (B) Sequence alignment of the mouse (amino acids 247–406) and human EpoR sequences (amino acids 248–407). Identical amino acids between the mouse (*M*) and human (*H*) EpoRs are indicated by asterisks (*). Important motifs for EpoR signaling (JM, juxtamembrane domain; Box1 and Box2, highly conserved sequence among cytokine receptors) and the phosphorylated tyrosine residues (Y343 and Y401) on mouse EpoR are illustrated. (C) Semi-quantitative RT-PCR analysis of the transgene expression in the spleens of 3 independent transgenic mouse lines. Expression of the *G1HRD-idEpoRic* transgene was detected by 27, 30, 33, 36, and 39 PCR cycles (left to right). HPRT was used as an internal control. (D) Membrane-associated localization of idEpoRic was observed in the spleen section of the line A transgenic mouse using laser confocal microscopy (left). A spleen section of a *G1HRD-GFP* transgenic mouse, which expresses GFP throughout the whole cell, is also shown as a control (right). The scale bar indicates 10 μm.

Using the chemically inducible homodimer system, we first generated a transgene expressing idEpoRic (inducible dimerization of EpoR intracellular domain), which consists of a myristoylation signal peptide, 2 CID-binding proteins (DmrB), the mouse EpoR intracellular subdomain (amino acids 247–406), and GFP (Fig. 1A) under the control of *G1HRD*, which allows expression in erythroid cells [21, 25]. The transgenic product was expected to associate with the cellular membrane through the myristoylation signal sequence (Myr) and to reversibly transduce growth signals through CID-mediated homodimerization of DmrB (Fig. 1A) [26]. EpoR(247–406), which contains the Box1, Box2, and juxtamembrane (JM) domains as well as 2 phosphorylatable tyrosine residues (Y343 and Y401), is highly conserved between humans and mice [27], demonstrating its functional importance in Epo-EpoR signaling (Fig. 1B).

Upon microinjection of the transgene construct into fertilized eggs, we obtained 3 independent lines (lines A, B, and C) of *G1HRD-idEpoRic* transgenic mice. Semi-quantitative RT-PCR analysis of the spleen demonstrated that the transgene expression levels of lines A and B were approximately 512- and 8-fold higher than that of line C, respectively (Fig. 1C). The differences in the expression levels were likely due to chromosomal position effects of the transgene integration sites [28].

Transgene-derived GFP expression was observed in 8% and 1% of the bone marrow MNCs by flowcytometry in lines A and B, respectively. However, GFP expression was not detected in the spleen or bone marrow of line C mice. These GFP expression data agree with the RT-PCR data (Fig. 1C). More than half of the GFP-positive cells in the bone marrow and fetal liver tissue of line A mice were distributed in the Ter119-positive erythroblastic cell fraction, and the remainder were found in other *Gata1*-expressing fractions, the majority of which were hematopoietic progenitors (below). GFP expression was not detected in the Mac1-positive or B220-positive bone marrow cells of line A mice. This GFP expression profile of the transgene was comparable to the profiles of other transgenes driven by *G1HRD*, as reported in our previous reports [21, 25]. We conclude that the transgenic expression of idEpoRic is fundamentally exclusive to the erythroid lineage cells.

The subcellular localization of the GFP-fusion idEpoRic was investigated in splenic sections of line A transgenic mice using laser scanning confocal microscopy. As expected, membrane-associated green fluorescent signals were observed in cells in the red pulp regions of spleens from *G1HRD-idEpoRic* mice, whereas distribution of the GFP signal was detected throughout the cell in the erythroid cells of the *G1HRD-GFP* transgenic mice expressing only GFP under the control of *G1HRD* (Fig. 1D). This result shows that the myristoylation signal sequence causes idEpoRic to associate with the cell membrane.

### CID dose and transgene expression level-dependent growth of the erythroid progenitors from *G1HRD-idEpoRic* mice

**Fig 2:**
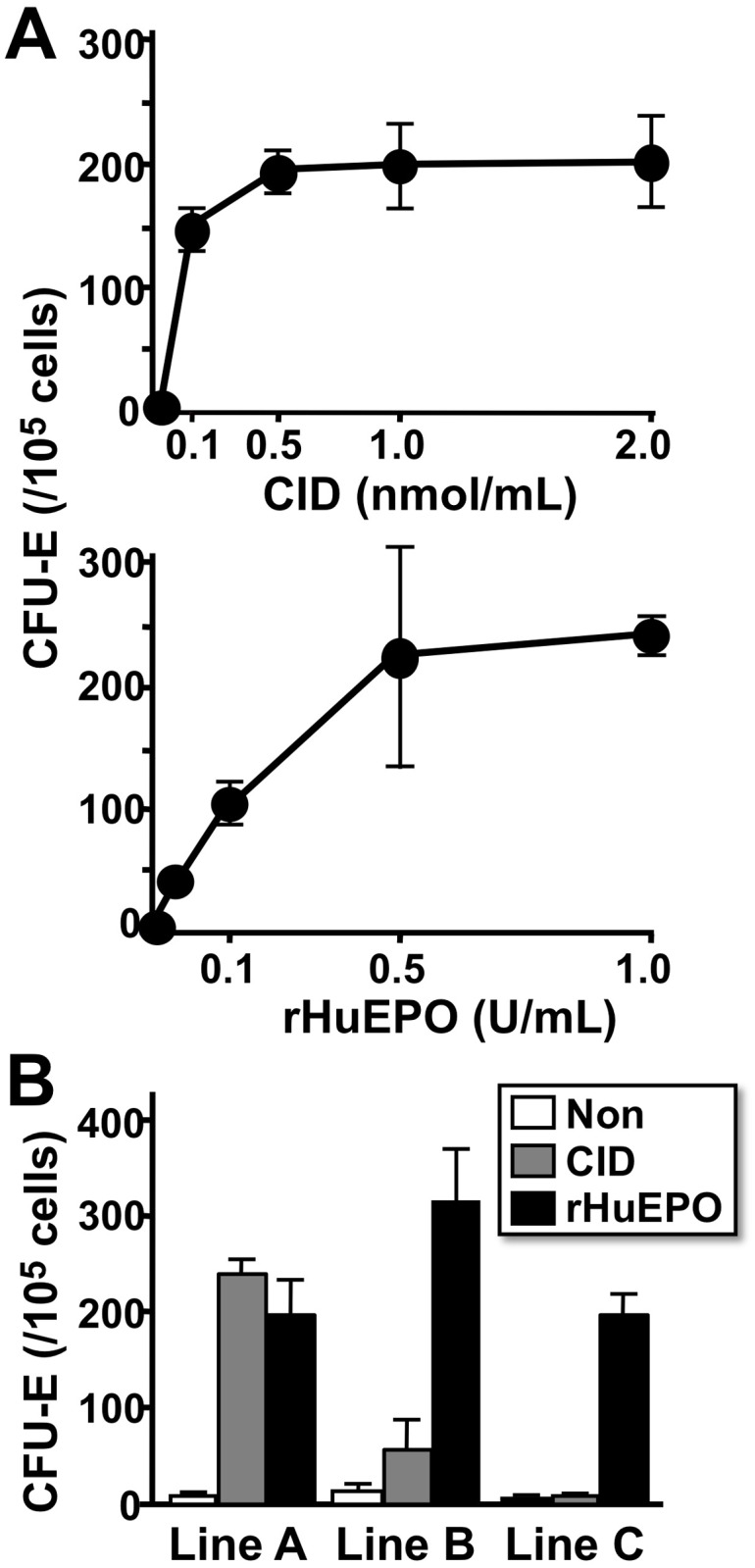
CID Dose- and Transgene Expression Level-dependent Colony Formation of *G1HRD-idEpoRic* Bone Marrow Cells. (A) Increased CFU-E—derived colony formation in response to incremental doses of CID or rHuEPO. Bone marrow MNCs from line A transgenic mice were used. (B) The bone marrow MNCs of 3 independent transgenic mouse lines were cultured in methylcellulose medium supplemented with CID (1.0 nmol/mL) or rHuEPO (2.0 U/mL) for 3 days, and the numbers of CFU-E—derived colonies were counted. Note that the numbers of CFU-E—derived colonies correlate tightly with the expression levels of the transgene (line A > line B > line C, see Fig. 1C). These assays were performed in triplicate and repeated 3 times. The results are shown as the mean ± standard deviations.

To test whether the CID-idEpoRic system recapitulates the Epo-EpoR system, a conventional erythroid colony-formation assay was performed with various concentrations of CID or rHuEPO. Increasing CID as well as rHuEPO doses resulted in an increased number of CFU-E−derived colonies in the line A bone marrow cells, and the colony counts reached maximum levels (approximately 200 colonies in 1.0 × 10^5^ cells) when the cells were incubated with 0.5 nmol/mL CID or 0.5 U/mL rHuEPO (Fig. 2A). These data show that the CID-idEpoRic system mimics the Epo-EpoR system in colony-forming assays and that the erythroid progenitors of the transgenic mice respond to CID in a dose-dependent manner.

Next, the effects of transgene expression on the sensitivities to CID were investigated by comparing the colony-forming activities of the 3 transgenic lines supplemented with 1.0 nmol/mL CID or 2.0 U/mL rHuEPO. In all transgenic mouse lines, approximately 250 colonies were counted in the 1.0×10^5^ bone marrow cells supplemented with rHuEPO, indicating no differences in the CFU-E progenitors among the transgenic mouse lines. Conversely, CID stimulation resulted in different numbers of colonies among the 3 lines. Because the colony numbers correlated with transgene expression levels (Fig. 2B), the sensitivity of the erythroid progenitors to CID was dependent on the transgene expression levels. These *in vitro* data indicate that the CID-idEpoRic system created using a transgenic strategy functioned similarly to the Epo-EpoR system in ligand dose- and receptor dose-dependent manners.

### Cell-lineage and subcellular distribution of idEpoRic in transgenic mice

**Fig 3:**
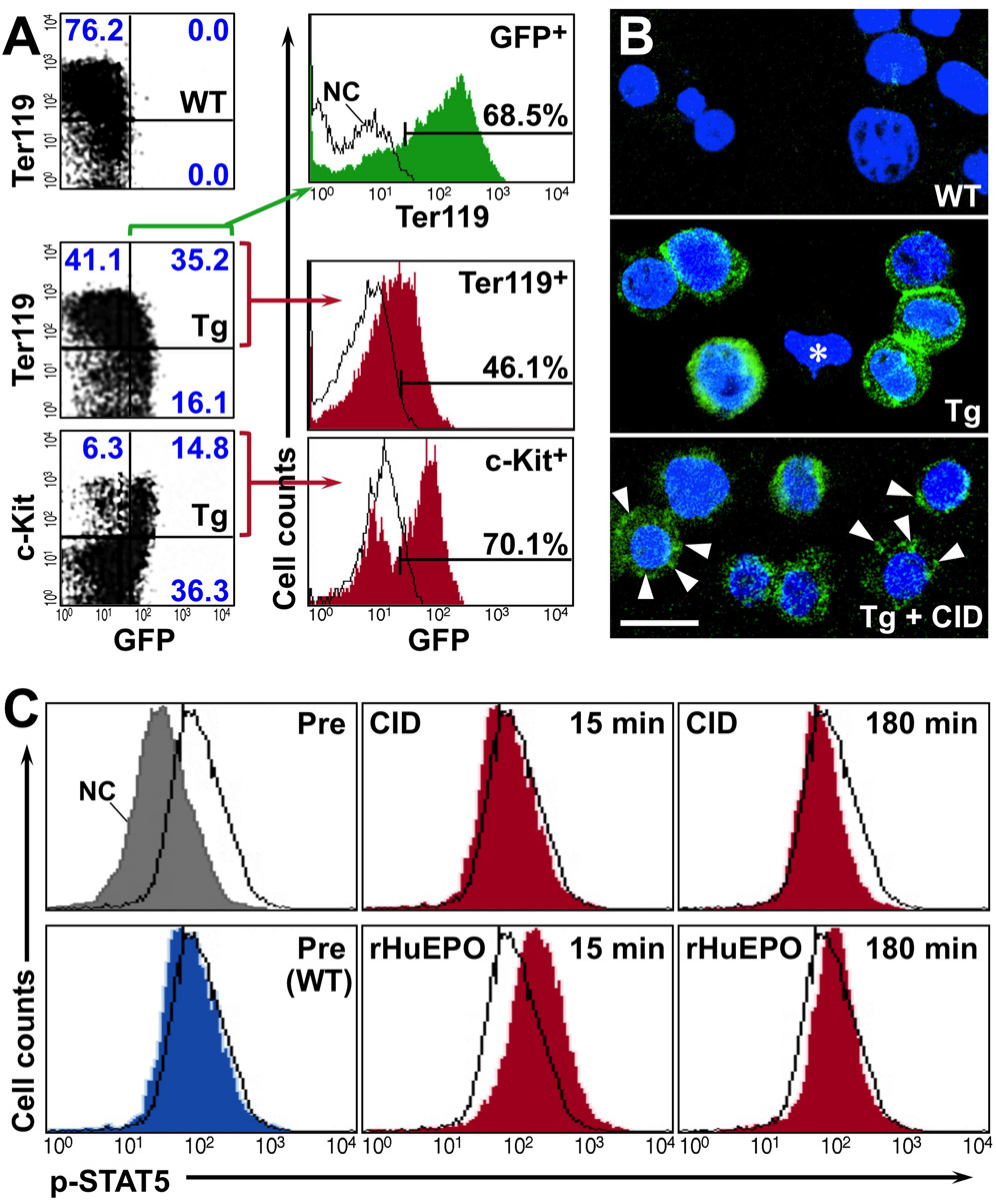
Changes in idEpoRic Localization and STAT5 Phosphorylation After CID Stimulation in Fetal Liver Hematopoietic Cells. (A) Flowcytometry of the fetal liver cells from wild-type (WT) or line A (Tg) mouse embryos at E12.5. Ter119 and c-Kit expressions levels were analyzed by GFP (idEpoRic) expression, and the percentages of cells in each quadrangle are shown (blue letters in dot plot data). The GFP^+^ fraction was analyzed using an anti-Ter119 antibody (green) or an isotype control antibody (as a negative control [NC], black line) in the top histogram. Cells positive for Ter119 or c-Kit have been analyzed by GFP expression, and black-line histograms show the data from wild-type littermates (middle and bottom histograms). The percentages of cells in the gated regions are shown in each panel. (B) The subcellular localization of idEpoRic in the E12.5 fetal liver was examined using laser confocal microscopy. Single cell suspensions from wild-type (WT) and transgenic (Tg) fetal livers at E12.5 were incubated with (bottom) or without (top and middle) 1 nmol/mL CID for 15 minutes, and the cells were observed after nuclear staining with Hoechst33342 (blue). Note the membrane-associated localization of GFP (green) in the transgenic cells (middle) and the internalization of the GFP^+^ clusters after CID stimulation (arrowheads). A GFP-negative cell (asterisk) is found in a preparation of the transgenic cells. The scale bar indicates 10 μm. (C) Intracellular phosphorylated STAT5 (p-STAT5) in E12.5 fetal liver cells was detected using PE-conjugated anti-phosphorylated STAT5(Y694)-specific antibodies. Cells from line A fetal liver were exposed to rHuEPO (2.0 U/mL) or CID (1.0 nmol/mL) for 0 (Pre), 15, or 180 minutes. The left panels show basal levels of p-STAT5 in the transgenic (black lines) and wild-type (WT, blue) cells compared with the isotype antibody controls (NC, gray).

The colony-formation assay (Fig. 2) also revealed that few non-erythroid lineage colonies were observed after 7 days of cultivation with CID. Since these benzidine-negative colonies were observed dishes without CID or rHuEPO, the formation of non-erythroid colonies was due to experimental background. Therefore, the CID-idEpoRic system driven by *G1HRD* appears to mainly stimulate erythroid colony-forming progenitors even though *G1HRD* is active in megakaryocyte, eosinophil and mast-cell lineages [21, 29–31]. Next, by taken advantage of the GFP-tagged idEpoRic, idEpoRic distribution was investigated in transgenic mice. The GFP fluorescence of *G1HRD-idEpoRic* transgenic mice was very weak compared with the fluorescence of *GIHRD-GFP* transgenic mice that we had previously established (Fig. 1D, 3A) [25]. Potentially, fluorescence may be weakened by fusion with artificial peptide sequences. Because flowcytometry of the line A mice showed that the E12.5 fetal liver contained many more idEpoRic (GFP)-expressing cells (approximately 50%) than the adult bone marrow or spleen (less than 10%, see below) in line A transgenic mice (Fig. 3A), fetal liver cells were used for molecular analyses.

The GFP^+^ cells were mainly distributed in the Ter119^+^ erythroid lineage (approximately 70%) or c-Kit^+^ progenitor cells (approximately 30%) (Fig. 3A). However, GFP signals were impossible to detect in the non-erythroid lineages, including megakaryocytes, eosinophils and mast cells. Non-erythroid GFP expression was most likely prevented because of the weak fluorescence intensity of idEpoRic. Most of the GFP-expressing fetal liver cells were Ter119-positive erythroblastic cells, and approximately half of the Ter119^+^ cells expressed the transgene (Fig. 3A). The cells in the c-Kit^+^ fraction were clearly divided into major GFP^+^ (70%) and minor GFP^-^fractions (Fig. 3A). This result indicates that idEpoRic expression was restricted to certain c-Kit^+^ hematopoietic progenitors, including erythroid progenitors.

Next, the dynamics of idEpoRic subcellular localization were analyzed using laser confocal microscopy after CID stimulation. The data showed that idEpoRic anchored to the cytoplasmic membrane appeared to undergo endocytosis by CID binding within 15 minutes (Fig. 3B). Some clusters of the internalized idEpoRic were observed (arrowheads in Fig. 3B). Endogenous EpoR was internalized soon after Epo binding through the FEGLFTTHK motif (amino acids 268–276, see Fig. 1B), which was included in the idEpoRic construct [32, 33]. Therefore, we suggest that the EpoR 160 amino acid domain used in the idEpoRic construct was sufficient for receptor endocytosis after ligand stimulation. Although it has been reported that 95% of endogenous EpoR molecules localize to the endoplasmic reticulum [13, 34, 35], most idEpoRic molecules are associated with the cell membrane, likely due to the myristoylation signal sequences.

The GFP foci were distributed not only around the cell membrane but also in the cytoplasm, and the total number of foci was more than 1,000 in each GFP^+^ cell from *G1HRD-idEpoRic* transgenic mice (Fig. 3B). This result suggests that more than 1,000 functional idEpoRic molecules exist in a cell because CID receptors are able to activate downstream signaling pathways after binding to CID anywhere in intracellular spaces [26]. There are approximately 1,000 functional Epo-binding sites (EpoR dimers) on the cell surface of erythroid progenitors, and this number decreases during erythroblast maturation [23, 36]. Thus, the number of functional idEpoRic molecules in a cell is likely greater than the number of EpoR molecules.

### Cell signaling of the CID-idEpoRic system

STAT5 phosphorylation is one of the most important signal transduction events following Epo stimulation [9, 37, 38]. We detected phosphorylated STAT5 (p-STAT5) in transgenic fetal liver cells by flowcytometry after *ex vivo* stimulation with CID or rHuEPO. Before stimulation, p-STAT5 accumulated in the hematopoietic cells of both wild-type and transgenic embryos at E12.5, which were exposed to endogenous Epo in the fetal liver (Fig. 3C, Pre). p-STAT5 levels were further increased by rHuEPO after 15 minutes (Fig. 3C). When the cells were incubated with rHuEPO for 3 hours, the enhanced phosphorylation was diminished (Fig. 3C). In contrast, CID did not change the p-STAT5 levels in the transgenic cells (Fig. 3C). We confirmed that CID induced STAT5 phosphorylation in cells transfected with idEpoRic expression vectors under culture conditions evicting Epo [16]. These observations suggest that CID-idEpoRic signaling could be insufficient for the additional accumulation of p-STAT5 in the primary cells constantly stimulated with endogenous Epo, which is locally secreted by hepatocytes in the fetal liver [39]. Neither rHuEPO nor CID changed the phosphorylation status of STAT1 in the fetal liver hematopoietic cells. Taken together, the CID-idEpoRic system functionally recapitulates the Epo-EpoR system in erythroid colony formation, but not in the *ex vivo* enhancement of STAT5 phosphorylation beyond endogenous Epo stimulation.

### CID induces *in vivo* erythropoiesis in a dose-dependent, lineage-specific, and reversible manner

**Fig 4:**
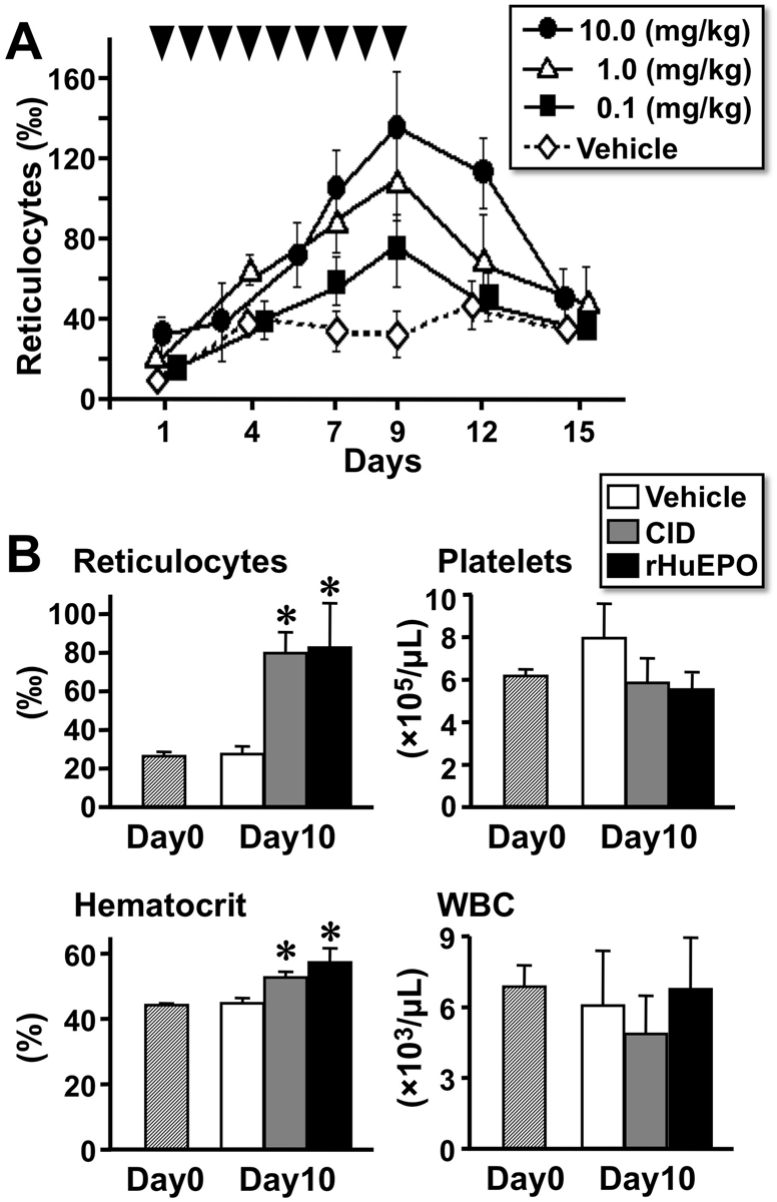
Dose-dependent, Reversible, and Erythroid Lineage-Specific Effect of CID on *In Vivo* Erythropoiesis. (A) Changes in the reticulocyte counts in the peripheral blood of the line A transgenic mice by intraperitoneal injection of CID (10.0, 1.0, or 0.1 mg/kg) or vehicle every day for 9 days (arrowheads). Reversible and dose-dependent induction of the reticulocyte counts by CID administration was observed. (B) The levels of reticulocytes, hematocrit, platelets, and white blood cells (WBC) in the peripheral blood of line A transgenic mice were counted before (Day0) and after (Day10) daily intraperitoneal injections of CID (10 mg/kg), rHuEPO (300 U/kg), or vehicle for 9 days (Days 1–9). These assays were performed in triplicate (3 mice for each group) and repeated 3 times. The results are shown as the mean ± standard deviations. *p < 0.01 compared with Day0 (hatched bars).

To investigate whether *in vivo* erythropoiesis is regulated by CID administration, the transgenic mice (line A) were intraperitoneally injected with CID for 9 consecutive days. Reticulocyte counts in the peripheral blood, which indicate erythropoietic activity, gradually increased in a dose-dependent manner during the repetitive CID injections and continued increasing with all injection doses until the CID injections were stopped on the ninth day (Fig. 4A). After the end of the CID injections, the reticulocyte number decreased to its initial levels within a week (Fig. 4A). These data from 3 mice per group demonstrate that CID injection enhances *in vivo* erythropoiesis in a dose-dependent and reversible manner.

High-dose (10 mg/kg) CID administration increased the reticulocyte counts approximately 3-fold on Day9 (Fig. 4A). One day after the 9 consecutive injections (Day10), the reticulocyte levels remained significantly higher than that of the vehicle controls (Fig. 4B). Because these injection schemes also significantly increased hematocrit levels (Fig. 4B), the CID-idEpoRic system effectively stimulates *in vivo* erythropoiesis. This increase was also observed when the mice were injected with a high dose (300 U/kg) of rHuEPO for 9 consecutive days (Fig. 4B) in our previous report [40]. Thus, 10 mg/kg (6.7 μmol/kg) CID was as effective as 300 U/kg rHuEPO *in vivo*, and 0.5 nmol/mL CID and 0.5 U/mL rHuEPO showed similar activity in the *in vitro* colony-formation assay (Fig. 2A). This result indicates that the *in vivo*: *in vitro* ratio of the effective concentrations was greater in CID (6.7 μmol/kg: 0.5 nmol/mL) compared with rHuEPO (300 U/kg: 0.5 U/ml). This difference may be because of the shorter *in vivo* lifetime of the CID molecule compared with rHuEPO.

**Fig 5:**
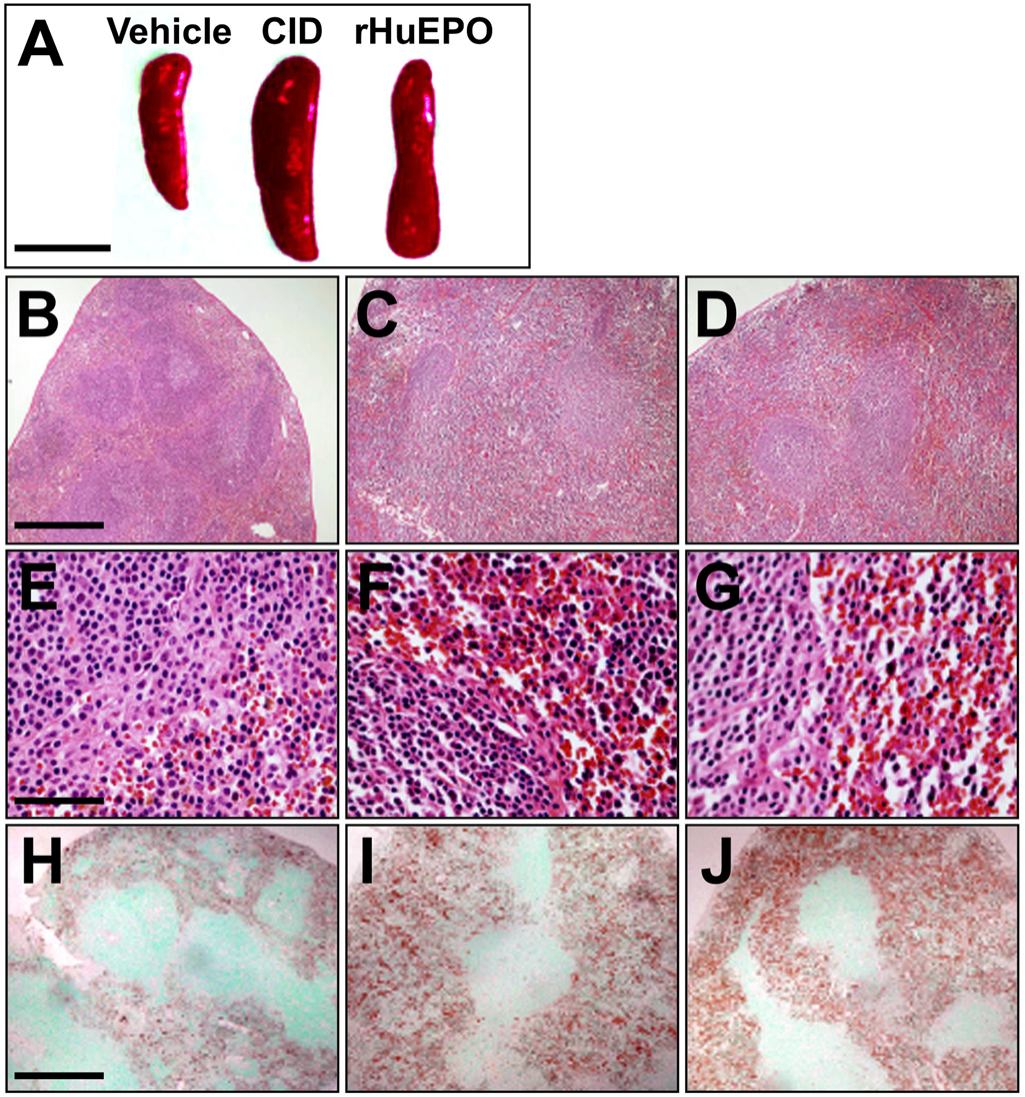
Enlargement of the Spleen Red Pulp by the CID-idEpoRic System. (A) Gross appearance of enlarged spleens after the injection of the vehicle, CID (10 mg/kg), or rHuEPO (300 U/kg) for 9 consecutive days. (B–G) Hematoxylin and eosin staining of the spleen sections from vehicle- (B, E), CID- (C, F), or rHuEPO-treated (D, G) line A transgenic mice. E, F, and G show higher magnification views. The enucleated red blood cells were observed in the spleen red pulps of CID- and rHuEPO-treated mice (F, G). (H–J) The spleen sections from vehicle- (H), CID- (I), or rHuEPO-treated (J) mice were stained with an anti-ß-globin antibody. ß-globin-positive (brown signals) erythroid cells were expanded in the spleens of CID- (I) or rHuEPO-treated (J) transgenic mice. The scale bars indicate 1.0 cm (A), 200 μm (B–D, H–J), and 80 μm (E–G).

To investigate the lineage specificity of the CID effects, the numbers of platelets and white blood cells in the peripheral blood were counted before and after CID injection. Neither CID nor rHuEPO caused significant changes in the concentrations of platelets or white blood cells on Day10 (Fig. 4B). Similar to rHuEPO injection, CID injection markedly enlarged the transgenic mouse spleens on Day10 (Fig. 5A). The red pulps positive for ß-globin staining were expanded and filled with mature, enucleated red cells in the CID- or rHuEPO-treated transgenic mice (Fig. 5B-J). The size of the white pulps negative for ß-globin staining was not altered by CID administration (Fig. 5H-J). These histological data from 4 mice in each group revealed that the CID-idEpoRic system exclusively stimulates erythropoiesis *in vivo*. We thus conclude that CID administration promotes erythropoiesis in *G1HRD-idEpoRic* transgenic mice but does not influence other cell lineages due to the specificity of the *G1HRD* activity in the erythroid cells.

### CID stimulates the proliferation and differentiation of idEpoRic-expressing erythroid progenitors

To further characterize the nature of CID signaling *in vivo*, we conducted a hematopoietic cell analysis. Flowcytometry was performed using the splenic MNCs of line A mice, exploiting several erythroid markers, including the transferrin receptor (CD71) and Ter119. A previous study reported that two-color staining of CD71 and Ter119 distinguishes erythroid cells [38]. CD71 is expressed at high levels by proerythroblasts and early basophilic erythroblasts, and these expression levels decrease during erythroid cell maturation, whereas Ter119 is expressed by terminally differentiated erythroblasts.

**Fig 6:**
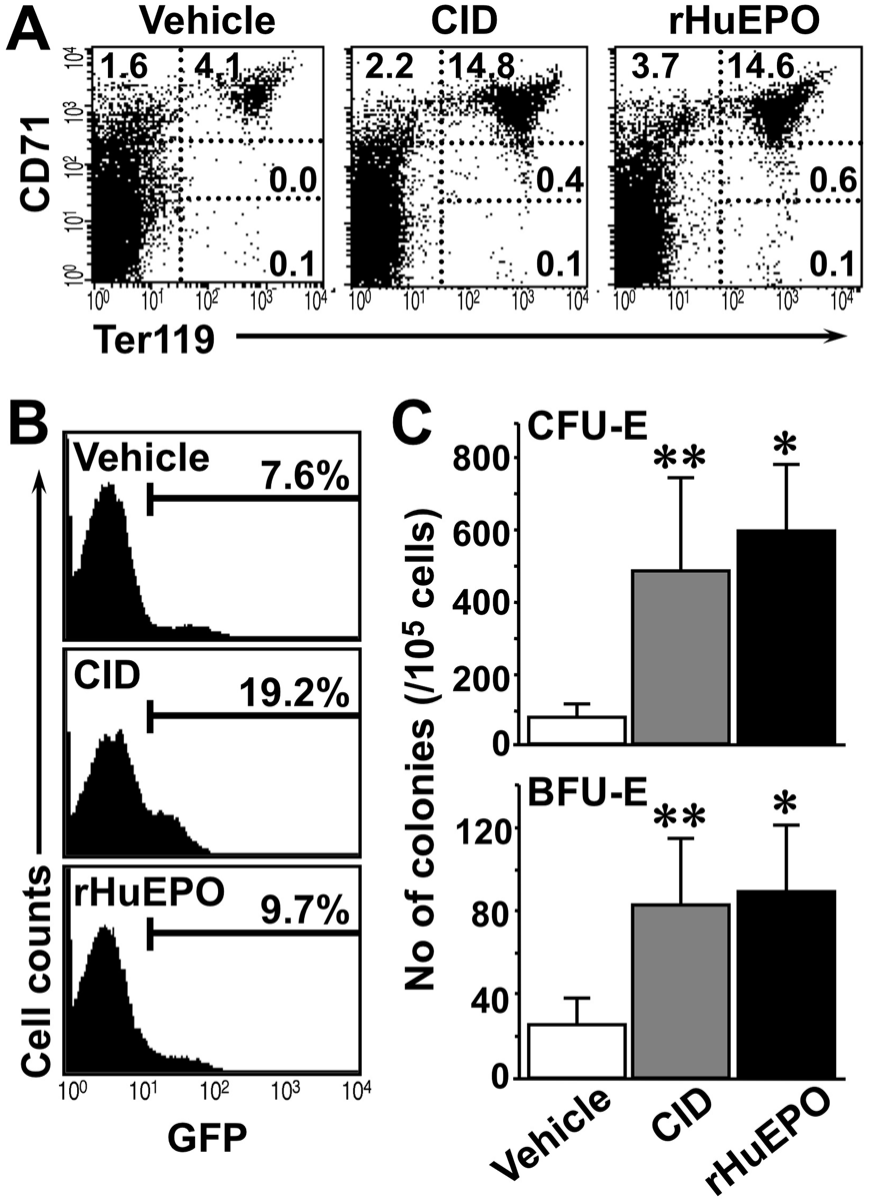
CID Stimulates the Growth of Erythroid Progenitors and Erythroblasts in the Spleens of Transgenic Mice. (A) CD71 and Ter119 were analyzed in splenic MNCs of line A transgenic mice by flowcytometry (upper panels) after the injection of vehicle, CID (10 mg/kg), or rHuEPO (300 U/kg) for 9 consecutive days. The erythroblastic fraction was separated into 4 maturation stages based on CD71 and Ter119 expression, and the percentages of cells in each region are represented in the graph. (B) GFP expression in splenic MNCs after the administration of CID or rHuEPO by the same strategy described for (A). CID exclusively induced GFP^+^ transgene-expressing cells, whereas a specific increase in GFP^+^ cells was not observed following rHuEPO administration. (C) The numbers of CFU-E and BFU-E erythroid progenitors were examined in the splenic MNCs of line A transgenic mice after the administration of vehicle (open bars), CID (gray bars), or rHuEPO (solid bars) by the same strategy described for (A). The results are shown as the mean ± standard deviations. **p < 0.05, *p < 0.01 compared with the vehicle-treated mice.

CD71^high^Ter119^high^ cells (basophilic erythroblasts), CD71^high^Ter119^low^ cells (proerythroblasts), and CD71^low^Ter119^high^ cells (orthochromatic erythroblasts) were increased in the spleens of the CID-treated and rHuEPO-treated transgenic mice on Day10 (Fig. 6A). Flowcytometry also showed that the number of GFP-positive cells increased significantly after CID injection but not after vehicle or rHuEPO injection (Fig. 6B). This result clearly indicates that CID exclusively acts on the cells expressing idEpoRic.

We then examined the numbers of CFU-E and BFU-E progenitors in the bone marrow of CID- or rHuEPO-treated transgenic mice. The CFU-E—derived colony number increased approximately 5-fold in the CID- and rHuEPO-treated mice compared with the control mice (Fig. 6C). The BFU-E colony number also increased in both the CID-treated and rHuEPO-treated mice (Fig. 6C). These results demonstrated that CID induced the proliferation and differentiation of erythroid progenitors, and this result is in good agreement with the function of Epo.

### Oral administration of CID to *G1HRD-idEpoRic* transgenic mice

Taking advantage of CID as a small chemical inducer, we examined erythropoietic induction after the oral administration of CID. Line A mice were treated with 10 mg/kg CID, the same dose as that used for peritoneal injection for 10 consecutive days. However, the reticulocyte counts did not increase significantly. Then, a 10-fold higher dose (100 mg/kg) of CID was perorally administered to the transgenic mice every day for 10 days, and hematopoietic cells were analyzed one day after the end of the dosing schedule.

Before the blood analyses, we checked assessed the effects of this high-dose, daily CID administration. The appearance and body weight did not change during oral administration of 100 mg/kg CID for 10 consecutive days, and a biochemical test of the serum samples showed that the daily administration of high-dose CID for 10 days did not cause any critical side effects in the liver, kidneys, or metabolic functions in the 4 mice examined (Table 1).

**Table 1:** Blood Biochemical Profiles after Oral Administration of CID.

	WT-Vehicle	Tg-Vehicle	WT-CID	Tg-CID
Total protein (g/dL)	5.6 ± 0.3	5.6 ± 0.2	5.4 ± 0.4	5.7 ± 0.4
AST (U/L)	59.3 ± 22.1	68.0 ± 12.3	52.0 ± 4.3	59.7 ± 11.8
ALT (U/L)	25.8 ± 9.7	26.5 ± 13.0	25.5 ± 11.0	23.0 ± 7.5
Total bilirubin (mg/dL)	0.42 ± 0.21	0.57 ± 0.21	0.47 ± 0.23	0.37 ± 0.09
BUN (mg/dL)	24.0 ± 5.4	18.9 ± 1.3	27.2 ± 0.6	19.0 ± 2.0
Creatinine (mg/dL)	0.37 ± 0.12	0.40 ± 0.08	0.53 ± 0.14	0.43 ± 0.12
Total cholesterol (mg/dL)	108 ± 24	107 ± 15	114 ± 10	104 ± 10
Blood sugar (mg/dL)	181 ± 37	139 ± 18	180 ± 9.0	162 ± 21

Biochemical indices of the *G1HRD-idEpoRic* transgenic mice (Tg, line A) were measured 1 day after oral administration of 100 mg/kg CID for 10 consecutive days. As controls, age-matched (8–16 weeks of age) wild-type (WT) and vehicle-treated mice were used. The values represent the mean ± standard error of 4 mice for each group. AST, asparagine transferase; ALT, alanine transferase; BUN, blood urea nitrogen.

**Fig 7:**
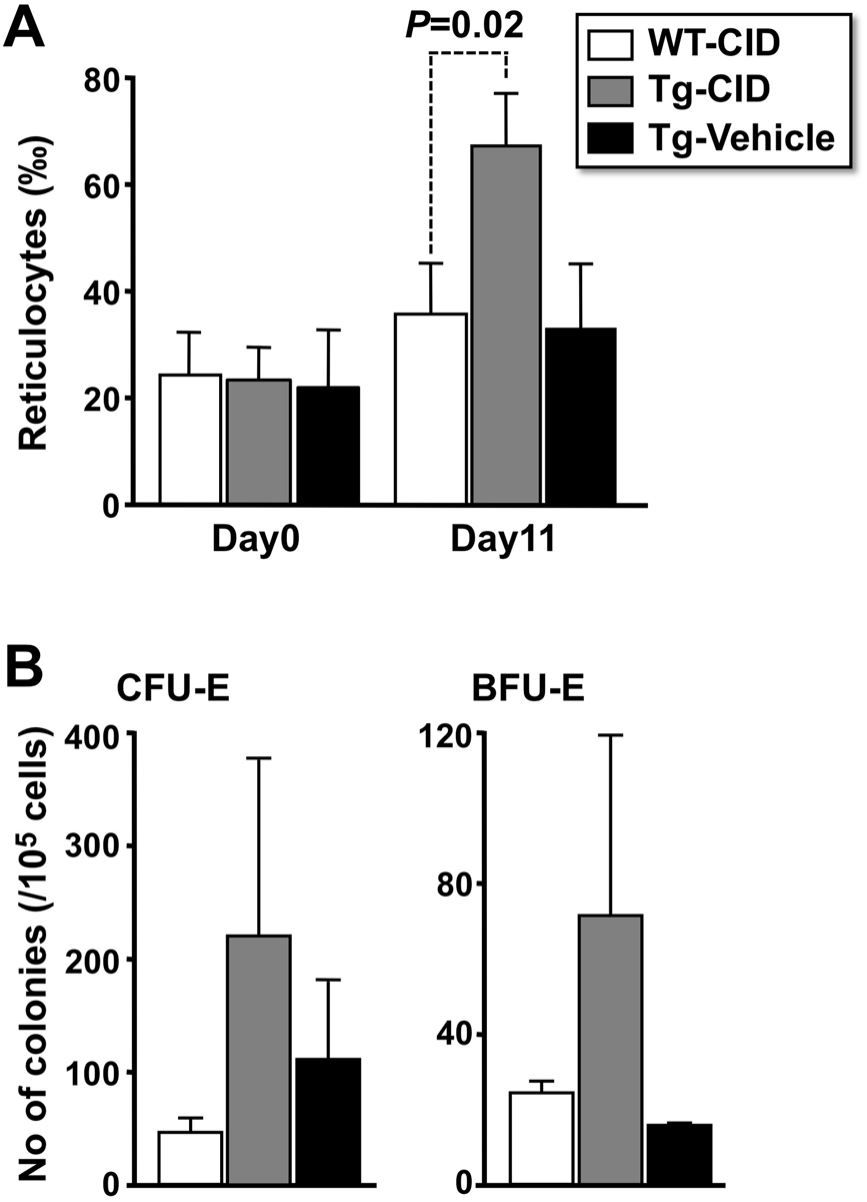
Oral Administration of CID Stimulates Erythropoiesis. (A) Reticulocytes in the peripheral blood of wild-type (WT) and transgenic (Tg) mice were counted before (Day0) and after (Day11) daily oral administration of CID (100 mg/kg) or vehicle for 10 days (Day1–Day10). (B) The numbers of CFU-E and BFU-E erythroid progenitors were also examined in splenic MNCs on Day11. Open bars indicate CID-treated wild-type mice (WT-CID). Gray and solid bars indicate CID- and vehicle-treated transgenic mice, respectively (Tg-CID and Tg-Veh). These assays were performed in triplicate with 4 mice for each group. The results are shown as the mean ± standard deviations.

A significant increase in reticulocyte count was observed compared with the reticulocyte number of CID-treated wild-type mice and vehicle-treated transgenic mice 1 day after daily administration of CID for 10 days (Day11, Fig. 7A). We also examined the numbers of CFU-E and BFU-E erythroid progenitors in the bone marrow on Day11. The number of progenitors in CID-treated transgenic mice was increased compared with those of CID-treated wild-type mice or vehicle-treated transgenic mice, even though significant differences were not observed (Fig. 7B).

## Discussion

Cytokines play key roles in the determination of cell fate. Most cytokines bind to their specific receptors forming homodimers on the cell surface, and initiate signal transduction by inducing a conformational change in the homodimers due to the close proximity of their intracellular domains [9–12]. In this study, we established a transgenic mouse model in which erythropoiesis was reversibly controlled by the administration of a small molecule CID, which induced the dimerization of a 160 amino acid sequence derived from the EpoR intracellular domain. This regulated system of erythropoiesis worked as efficiently as the Epo-EpoR system *in vivo* and provided information regarding the possibility of a peroral strategy for anemia treatment combined with gene therapy.

The Epo-EpoR system is indispensable for the proliferation and differentiation of erythroid cells [2]. Indeed, kidney diseases often cause chronic anemia due to insufficient Epo production from damaged REP cells [3–5]. rHuEPO has been used to treat renal anemia in patients with chronic kidney diseases for more than 20 years [1, 7]. rHuEPO is also effective for myelodysplastic syndrome, chronic disease-related anemia, cancer chemotherapy-related anemia, ß-thalassemia, and prolonged anemia following allogenic hematopoietic stem cell transplantation [1, 7]. Thus, rHuEPO is highly expected to be suitable because of its broad efficacy in many clinical situations. However, the administration routes of rHuEPO are limited to subcutaneous or intravenous injection, and its side effects, including hypertension, seizures, hyperkalemia, and hypercoagulable states, are distressing to patients [1, 7]. Here, we demonstrate that CID permits the control of erythropoiesis through the activation of EpoR signaling in mice expressing a CID-responsive transgene under the control of the *G1HRD* erythroid gene regulatory sequences. The CID-mediated dimerization of chimeric EpoR (idEpoRic) reversibly activates erythropoiesis *in vivo*. We believe that it may be possible to treat anemic patients by gene therapy through the oral administration of CID instead of by Epo injection without the rHuEPO side effects.

CID-mediated homodimerization systems have previously been investigated *in vivo*. For instance, Mpl dimerization induces a cell growth switch *in vivo*, and clinical trials investigating CID (AP1903)-mediated caspase-9 dimerization have been conducted to induce apoptosis of donor T cells, which attack host cells and cause graft-versus-host disease after stem cell transplantation [16–20, 41]. However, previous studies have not clearly investigated cell lineage-specific regulation by CID systems because CID has targeted a wide range of cells due to the expression of the DmrB-fusion proteins from retroviral vectors. To establish a lineage-specific regulatory system, this study used the *G1HRD* promoter for the transgenic expression of idEpoRic. When using the CID-idEpoRic system with *G1HRD*, CID exclusively induces the proliferation and differentiation of erythroid lineage cells, including immature progenitors and differentiated erythroblasts. This result indicates that the *G1HRD-idEpoRic* transgene allows for the exclusive action of CID in erythroid lineage cells. We have identified the minimal functional elements in the 8-kb *G1HRD* fragment, and the total length of the *miniG1HRD* cassette is less than 1 kb [42]. Recent studies have developed viral vectors containing tissue-specific gene promoters [43]. Therefore, it is possible to expect that the *miniG1HRD-idEpoRic* gene cassette may be virally delivered into patient hematopoietic stem cells to treat anemia with oral CID administration instead of Epo injection.

We generated 3 lines of transgenic mice expressing the *G1HRD-idEpoRic* transgene at different levels. The erythroid colony-formation assay revealed that sensitivity to CID is dependent on the transgene expression level. This result is comparable to our previous report using the *EpoR*-gene-modified mice, which showed that the number of cell surface receptors determines the sensitivity to their ligands [23]. CID-peritoneal injection demonstrated that CID reversibly induces erythropoiesis in a CID dose-dependent manner. These features of the CID-idEpoRic system are identical to those of the Epo-EpoR system.

The sufficiency of a 160 amino acid sequence of the EpoR intracellular domain (amino acids 247–406) for *in vivo* erythropoiesis indicates that the domain is minimally essential for the EpoR signaling pathway under the chemically inducible dimerization system. Indeed, a 148 amino acid sequence (amino acids 225–372), including a 23 amino acid transmembrane domain, has been shown to be the minimal essential domain for *in vivo* EpoR signaling [24]. The close proximity of the EpoR intracellular domains triggers the activation of JAK2 followed by STAT5, which leads to the phosphorylation of the 8 tyrosine residues in the EpoR intracellular domain and the recruitment of multiple signaling molecules [9]. The embryonically lethal anemia observed in both JAK2- and STAT5-deficient mice, which resemble Epo- or EpoR-deficient mice, demonstrates the crucial roles of JAK2 and STAT5 in Epo-dependent erythropoiesis [2, 38, 44]. However, mutation of all 8 tyrosines in the EpoR intracellular domain in mice causes extremely minor abnormalities in steady-state erythropoiesis [45]. This result may indicate that additional proteins, such as docking molecules, complex with EpoR to create a signaling platform for erythropoiesis.

Taken together, this study proposes a chemically regulated system of erythropoiesis that can be used as part of a peroral therapeutic strategy to treat Epo-deficiency anemia, and this study provides us with the molecular mechanisms explaining the activation of the Epo-EpoR system through the conformational change of the EpoR homodimer.
